# Characterization of antibodies for quantitative determination of spiggin protein levels in male and female three-spined stickleback (Gasterosteus aculeatus)

**DOI:** 10.1186/1477-7827-7-46

**Published:** 2009-05-14

**Authors:** Håkan Berg, Nikolai Scherbak, Harri Liimatta, Erik Hoffmann, Johnny Karlsson, Per-Erik Olsson

**Affiliations:** 1 Örebro Life Science Center, School of Science and Technology, Örebro University, SE-70182 Örebro, Sweden; 2 Department of Zoology, Stockholm University, SE-10691 Stockholm, Sweden

## Abstract

Spiggin is an adhesive glycoprotein produced in the kidney of sticklebacks during the breeding season and is subsequently secreted into the urinary bladder from where it is employed for nest building. Since the production of the protein has been shown to be under androgenic control, spiggin has been suggested to be a useful biomarker for androgenic substances in the environment. In this study, two polyclonal spiggin antibodies based on synthetic peptides and one polyclonal antibody directed against native spiggin have been characterized. The antibodies ability to identify spiggin was investigated by quantitative immunoassay. For both peptide antibodies the quantification range was determined to be between 1 and 80 ng spiggin and determination of renal spiggin levels from immature and mature males displayed a 15-fold increase in total spiggin content of the kidney resulting in a 6-fold increase in male kidney weight due to hypertrophy. The kidney somatic index (KSI) was found to correlate well with the total renal spiggin content and therefore it appears that KSI in sticklebacks could be used as an initial method to identify substances displaying androgenic effects. Furthermore, western blot analysis revealed that the polyclonal antibodies recognize different spiggin isoforms and that spiggin can be detected in the urinary bladder and kidney of both males and female sticklebacks. In order to develop a quantitative detection method for native spiggin it is necessary to produce a standard that can be used in a bioassay. Due to the adhesive and polymerization characteristics of spiggin the protein is difficult to use as a standard in bioassays. So far spiggin has been shown to exist in at least 14 isoforms, all of which contain polymerization domains. To overcome the solubility problem we have produced recombinant spiggin gamma, with only one polymerization domain, that can be expressed in E. coli. Western blot analysis demonstrated that the polyclonal antibodies were able to detect recombinant spiggin gamma protein in bacterial cell lysate, suggesting that it may be developed into a useful source of standard spiggin to be used for quantitative determination of androgen induced spiggin production in sticklebacks.

## Background

Three-spined stickleback (*Gasterosteus aculeatus*) are small fish (~10 cm) with three spikes on their back and two abdominal spikes that are widely distributed throughout the northern hemisphere, and live and reproduce in fresh, brackish and salt water.

During the reproductive season, androgens control the development of male stickleback secondary sexual characteristics such as blue eyes, a red belly and hypertrophied kidneys. Androgens has also been suggested to initiate male reproductive behavior in sticklebacks, such as territorial establishment and nest building using a combination of plant fragments and renal secretions made up of an adhesive glycoprotein called spiggin [1].

Spiggin is a glycoprotein complex consisting of a multitude of isoforms, which are formed by alternative splicing, and their expression is regulated by 11-ketoandrogens [2]. Structural analysis of the spiggin subunits shows that the protein is highly hydrophobic and thereby insoluble in water. Due to its regulation by androgens, spiggin has been suggested to be a valuable biomarker for environmental androgen and antiandrogen exposure and the three-spined sticklebacks abundance makes it a good candidate as a species for environmental monitoring of androgenic substances. Sticklebacks are easy to keep and breed, which make sticklebacks a good choice as a monitoring species for mechanisms and effects of androgenic exposure. [3].

Spiggin is synthesized from at least 5 gene-loci and multiple subunits occur as a result of alternative splicing [4]. Initially we identified three spiggin subunits, α, β and γ [2], but recently, it has been shown that spiggin is encoded by a multi-gene family that give rise to at least 14 protein isoforms. This is suggested to contribute to the effective synthesis of large amount of the glue-like spiggin, during the sticklebacks reproductive season [5].

The spiggin isoforms all display amino acid sequence similarities to other adhesive proteins such as mucin from *Xenopus *(28%), rat (27%), human (27%) and to the human von Willebrand factor (vWF) protein D-domain (26%) [2]. The vWF D-domain plays an important role in protein multimerization and for the function of vWF in blood coagulation while mucin plays an important role in the protection of epithelial cells by creating a viscous surface cover [6]. Unlike the vWF and the vWF-related proteins, the D-domain within each subunit of spiggin is organized into non-tandem repeats separated by a number of cystein-rich regions [2]. The shortest of all spiggin subunits identified so far is spiggin γ which is 472 residues long and contain 2 D-like domains coupled by a cystein-rich region. Native spiggin protein is difficult to purify due to multimerization and its hydrophobic nature. Development of a recombinant spiggin protein would facilitate the production of spiggin standard to allow quantitative analysis of spiggin protein. Spiggin γ is a good candidate for the development of a recombinant spiggin protein as it is the smallest isoform and only has one polymerization domain (CGLCG) [2].

In the present study three polyclonal spiggin antibodies have been characterized and used to develop a quantitative immunoassay. The antibodies have been screened to determine which spiggin isoforms they recognized. In order to achieve knowledge of the basal expression level of spiggin in males and females spiggin levels in immature and mature fish of both sexes were determined. Furthermore, the uses of recombinant spiggin γ protein, as a standard for spiggin bioassays were examined.

## Methods

### Polyclonal antibody production

Synthetic peptides KTK (KTKEIQTYTCRTFGS-C), corresponding to amino acid 24 to 38 in the three-spined stickleback spiggin protein (Jones *et al*., 2001) and HRD (HRDELIRDSKLHDHR-C) corresponding to amino acid 173 to 188, were produced by Innovagen AB (Lund, Sweden). The polyclonal antisera against subunits KTK and HRD of spiggin were produced by Agrisera AB (Vännäs, Sweden).

### Experimental animals and samplings

Adult three-spine sticklebacks were caught in Oresund and were kept in a 700 L tank with brackish water (0.5% salinity) at low temperature (4–7°C), short photoperiod of 8 hrs light and 16 hrs darkness (L:D 8:16) to maintain them in non breeding condition. In order to induce maturation, the sticklebacks were transferred to 200 L aquaria and held under long photoperiod L:D 16:8 which resulted in visible secondary sexual characteristics in males. Prior to sampling the fish were euthanized by a swift strike to the head, weighed and bladders and kidneys were excised and weighed. The excised tissues were immediately transferred to 2.0 ml Eppendorf tubes (Eppendorf, Germany), containing 400 μl of an ice cold 10% saline solution, homogenized, and kept on ice or at 4°C until analyzed.

### 11-Ketotestosterone induction

For the spiggin induction studies, non breeding stickleback (1.39 ± 0.3 g) were injected intraperitoneally with 5 μg 11-ketotestosterone (11-KT) per kg bodyweight. For the negative control, carrier alone (peanut oil) was injected. Following the injection the fish were kept under normal conditions for 72 hours before sampling. At sampling the stickleback were sacrificed, weighed and the kidneys were excised and immediately homogenized in an ice cold 10% saline solution prior to performing an ELISA.

### ELISA procedures

ELISA was performed using NUNC MaxiSorp 96-well plates, (Nunc A/S, Denmark), synthetic peptides were diluted to a final concentration of 20 ng in 100 μl coating buffer (0.1 M Na_2_CO_3_, pH 9. 6). The plates were incubated at room temperature (RT, 22°C) for 1 hr followed by a 3 × 3 minutes wash with 200 μl PBST (0.05 M phosfate buffered saline pH 7.4 with 0.5% Tween 20) and subsequent addition of 200 μl 5% dry milk dissolved in PBS, for 1 hour at RT. The plate was subsequently washed with PBST, 3 × 3 minutes before addition of 100 μl primary antibody to each well and incubated at RT for 1 h. Following the incubation the plate was washed with PBST, 3 × 3 minutes prior to addition of a HRP conjugated secondary antibody, anti-rabbit Ig (Amerersham, England, 1:2000). The plate was incubated at RT for 30 minutes and followed by a 3 × 3 minutes wash in TBST. Detection was enabled by addition of 100 μl 2,2'-azino-bis (3-ethylbenzthiazoline-6-sulfonic acid (ABTS, Pierce, Rockford, IL, USA), absorbance was measured with a plate-reader, (TD-20/20 luminometer, Turner designs), at 405 nm after 60 minutes. The linear range of detection was calculated and used for measurements on serial dilutions of the peptides.

### Protein concentration determination

Protein concentration were determined by using the Bio-Rad protein assay, (Bio-Rad, USA), according to the manufacturers instructions. Briefly, a standard dilution series of bovine serum albumin (BSA) was made in PBS, and Bio-Rad protein assay reagent (Bio-Rad, USA), diluted 1:3 in PBS, was used as detection reagent. Absorbance was measured in a plate-reader at 595 nm.

### Western blot procedures

SDS-PAGE was performed using a 5% (w/v) stacking gel and a 7.5% (w/v) separating gel. Prior to electrophoresis the samples were mixed with sample buffer and heated at 65°C, 10 minutes and immediatley put on ice. 6 μg of total protein was added to each well. Electrophoresis was carried out at 145 V, 45 minutes at RT. Following SDS-PAGE, the separated proteins were electroblotted onto PVDF membranes (Hybond-P, Amersham, England). Electro-transfer of proteins was performed overnight at 5°C, 30 mV in a regular transfer buffer containing 20% methanol. Upon completion of transfer, the membranes were incubated in tris buffered saline (TBS) containing 5% dry milk for 2 hours. The membrane was rinsed in TBST (TBS with 0.1% (v/v) Tween 20) for 3 × 3 minutes prior to a 1 hour incubation in primary antibody. Subsequently the membranes were washed 3 × 15 minutes in TBST and incubated with the secondary antibody, diluted 1:3000 in TBST, for 30 minutes. Prior to detection the membrane was washed, 3 × 15 minutes in TBST. The detection was made by using ECL (Amersham, England), according to the manufacturers instructions.

### Cloning and transformation

The open reading frame (ORF) of spiggin γ was amplified from a pBK-CMV-spiggin γ vector construct using the following PCR (primers: forward – 5'-CAC CAT GAC AAC CCA GCG ATG GAT TC-3'; reverse-5'-CGG TGT TTC TGG GTT ATC AC-3') using proof-reading VENT DNA-polymerase (New England Biolabs, USA). Optimized PCR conditions were as follows: 97°C for 3 minutes → [95°C for 30 seconds, 55°C for 30 seconds, 72°C for 3 minutes] × 35 cycles → 72°C for 10 minutes. Following DNA electrophoresis the PCR product was isolated from the gel using QIAquick Gel Extraction Kit (Qiagen, Germany). TOPO-TA directional cloning reaction into pET101 vector, (Invitrogen, USA), and further transformation into Top10 *E. coli *strain, (Invitrogen, Carlsbad, CA, USA) were performed according to the manufacturer's manual. For the over-expression purpose the purified pET101/spiggin γ vector was transformed into BL21o (DE3) pLysS *E. coli *(Invitrogen, USA). Transformed bacteria were grown on LB plates containing 50 mg/L carbenicillin. Screening of the colonies was performed using PCR under the previously mentioned conditions using forward T7 and spiggin reverse primers pair.

### Protein over-expression and bacterial cell lysis

20 μL of bacterial glycerol stock was added to 20 ml LB supplied with 50 mg/L carbenicillin and cultivated on shaker at 37°C. Protein expression was induced by addition of 1 mM IPTG at OD_600 _= 0.8. Following overnight incubation on shaker at 30°C, the bacteria were pelleted by centrifugation for 20 min at 4000 g. Pelleted bacterial cells were lysed by the addition of 600 μL of CelLytic B 2× buffer, (Sigma, USA), supplied with 20 mM 2-mercaptoethanol, DNase and Complete™ protease inhibitor cocktail (Roche, Basel). In order to detect the over expressed protein the supernatant was mixed with Laemli loading buffer [7], boiled for 5 min and was resolved on a 12% SDS-PAGE prior to western blot analysis as described above. The immunodetection was performed using a polyclonal antiserum raised against N-terminal peptide of spiggin, or the polyclonal antiserum against native spiggin protein (1:5000 working dilution), alternatively – the mouse monoclonal anti-polyhistidine antibody (1:2500 dilution; Sigma-Aldrich, St. Louis, MO). Proteins were visualized using Western Blue^® ^Stabilized Substrate for Alkaline Phosphatase (Promega, USA).

### Statistics

Data are presented as mean ± standard deviation and all statistical analysis were performed with Graphpad Prism V 3.03 (GraphPad Software, USA). Statistical analysis of differences in spiggin levels was performed by one-way Anova, followed by Tukey's post hoc test.

## Results

**Figure 1:**
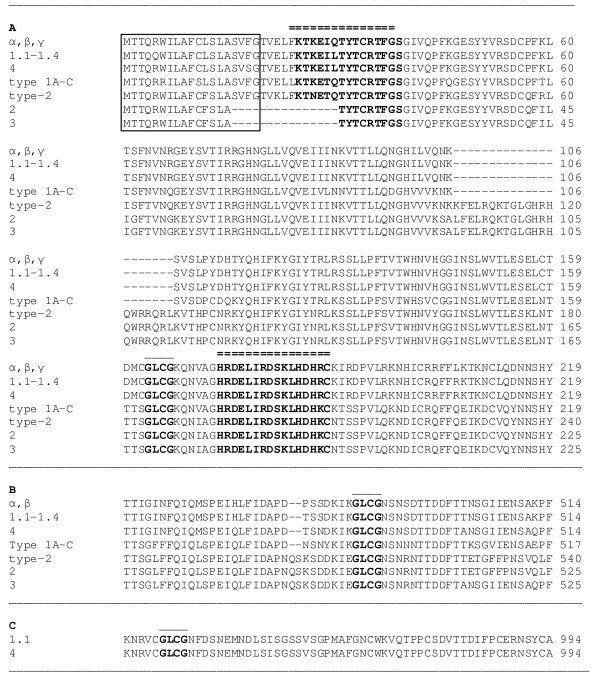
**Alignment of spiggin amino acid sequences**. A. Conservation of the N-terminal region containing the signal peptide (boxed), the sequences utilized to synthesize the KTK and HRD peptides (bold with double line above sequence) and the glycosylation sequence (bold with single line above sequence). B. The central region of all spiggin proteins, except spiggin γ, contains one polymerization site (bold with single line above sequence). C. The C-terminal region of Spiggin 1.1 and 4, contain one additional polymerization site (bold with single line above sequence). Spaces (-) are inserted to allow the alignment of homologous amino acids.

**Figure 2:**
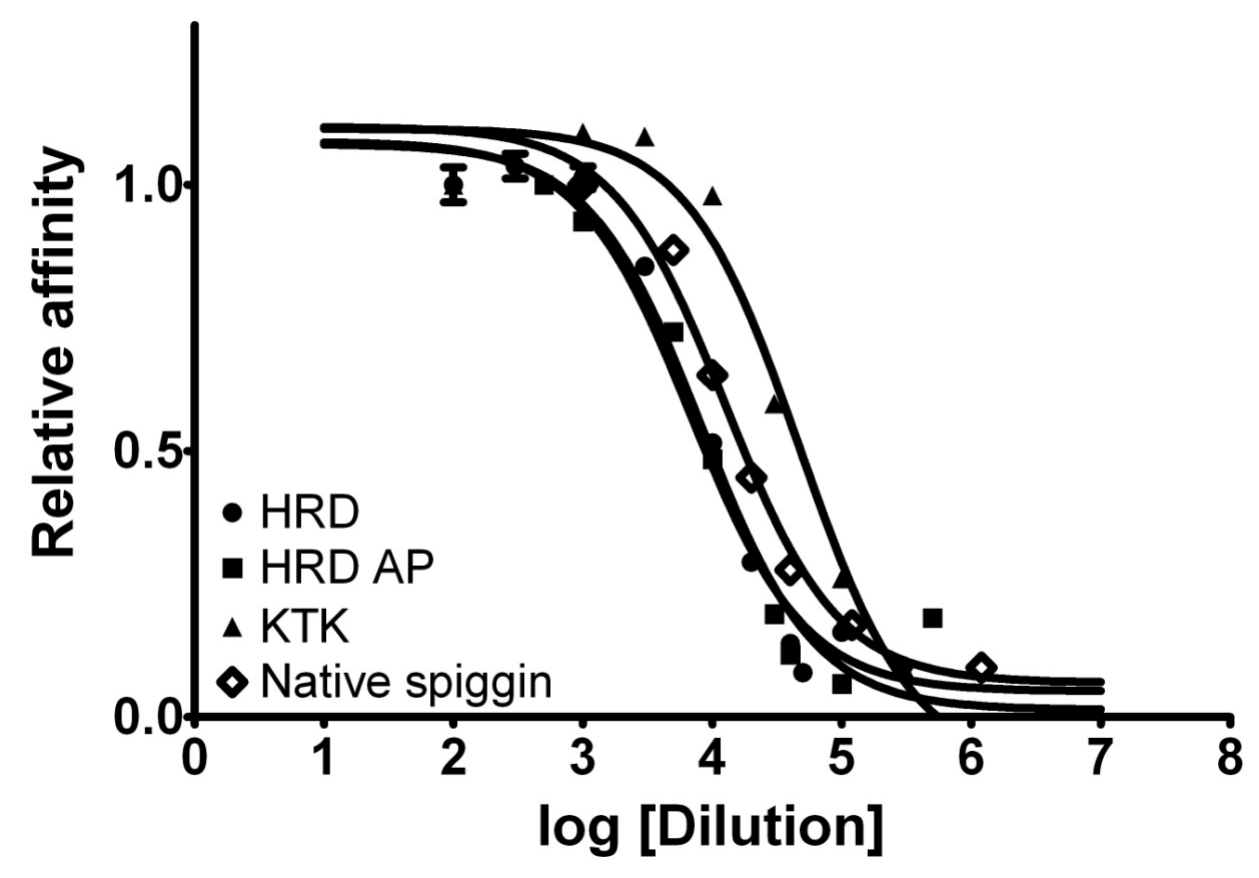
**Representative titration curves of antibody dilution curves**. For the peptide antibodies the curve was produced against known amounts of peptide while the native antibody was titrated against native spiggin. Each dilution was measured in triplicate and a relative value was calculated from the mean values of the lowest dilution.

**Figure 3:**
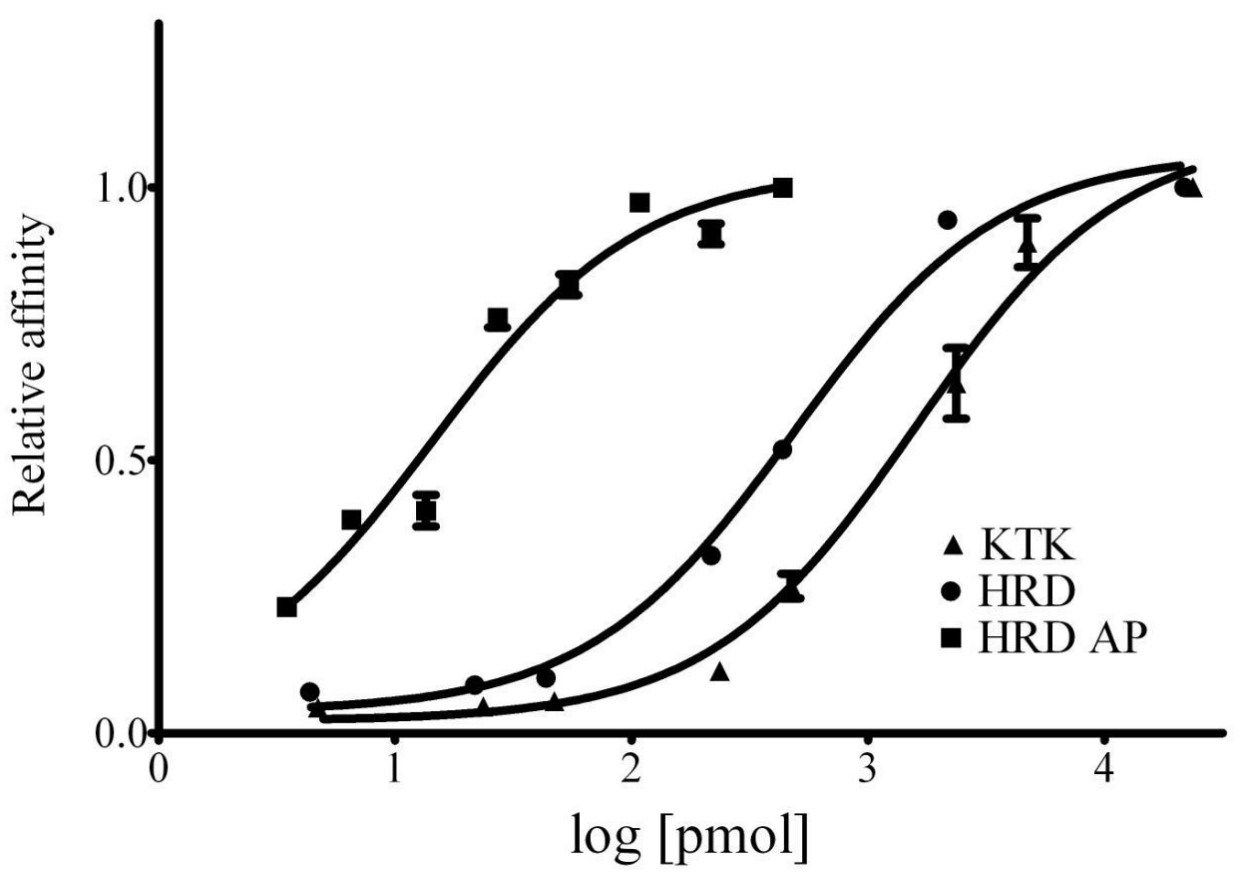
**Determination of linear detection range**. Serial dilutions of the HRD peptide in conjunction with HRD antibody diluted 1: 5000, HRD peptide in conjunction with affinity purified HRD antibody diluted 1: 10000, and KTK peptide in conjunction with KTK antibody diluted 1: 5000. Each dilution was measured in triplicate.

Native spiggin is a protein complex made up of a multitude of distinct subunits. Even though it has been shown that different isoforms are located in at least three different loci and that as many as 14 different isoforms exist, structural analysis reveal that all known spiggin isoforms share a high level of homology (Fig. 1). To establish the antibody titers and working range for the polyclonal antibodies, ELISA with serial dilutions of KTK, HRD, affinity purified HRD (HRD AP) and antibodies directed against native spiggin, were performed in a range from 1:100 to 1: 1000 000 using 20 ng of their respective synthetic peptide per well (Fig. 2). Even though the different antibodies displayed slight variation in detection limits all antibodies displayed linearity when diluted between 1:10000 and 1:40000. The obtained antibody dilution curves were used to determine the linear range of detection. Standard curves of the two peptide based antibodies, KTK and HRD, were produced using dilution series of synthetic peptides in a molar range of 5–20 000 pmol (Fig. 3).

**Figure 4:**
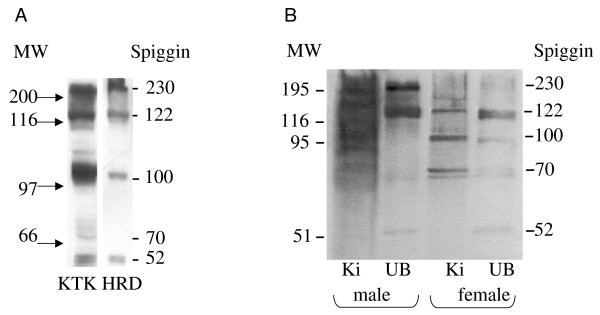
**A. Western blot analysis of kidney and urinary bladder content**. A. Kidney cytosol from male three-spined stickleback was used to determine the presence of spiggin isoforms using antibodies against KTK and HRD respectively. B. Western blot analysis of spiggin content of male and female three-spined stickleback kidney and urinary bladder using antibodies directed against native spiggin. The deduced molecular masses of different spiggin isoforms are indicated on the right. Ki = Kidney, UB = Urinary bladder. Molecular mass of the protein standard is indicated on the left (kDa).

As 14 spiggin isoforms have been reported, it was of interest to determine if bands corresponding to these subunits could be detected using the different spiggin antibodies. The kidney and urinary bladder was collected from male and female stickleback and used in Western blot analysis to investigate the specificity of the KTK and HRD antibodies (Fig. 4a), as well as the specificity of the polyclonal antibody directed against native spiggin in male and female kidney and bladder (Fig 4b). As shown in figure 4 there were bands corresponding to the sizes of all spiggin protein isoforms (as outlined in Table 1). An interesting observation was that the different antibodies, although all of them identifies spiggin protein, display different specificity toward the different isoforms. The KTK antibody can be expected to show low specificity towards spiggin 2 and 3 as six out of the 15 amino acids in the KTK peptide are missing from these isoforms (Fig. 1). In contrast, the HRD sequence is perfectly conserved in all spiggin proteins (Fig. 1).

**Table 1:** Spiggin protein isoforms. The calculated primary molecular mass and sequence similarity (%) to spiggin γ based on protein sequences obtained from the indicated GeneBank accession numbers are shown.

Spiggin genes	Calculated MW (kDa)	% similarity to spiggin γ	Accession number
1.1	118.6	97	BAE92619
4	118.6	97	BAE92625
α	102.7	100	AAK15297
1.2	101.9	97	BAE92620
2	101.0	73	BAE92623
1.3	99.9	97	BAE92621
3	98.4	71	BAE92624
Type 2	73.4	71	BAE48785
Type 1A	69.9	78	BAE48782
Type 1B	69.9	78	BAE48783
Type 1C	69.9	78	BAE48784
β	69.6	100	AAK15298
1.4	69.5	97	BAE92622
γ	54.5	100	AAK15299

**Figure 5:**
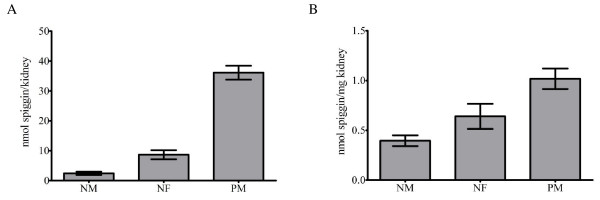
**Quantification of renal spiggin levels from non-spawning males and females and pre-spawning males using the HRD antibody**. A. Spiggin content in nmol per kidney. B. Spiggin levels in nmol per mg kidney.

The presence of spiggin was determined for non-spawning and pre-spawning three-spined stickleback. Determination of the relative kidney size (KSI, kidney somatic index) showed that the non-spawning males had a KSI of 0.48 while the non-spawning females had a KSI of 0.76. Pre-spawning males on the other hand had a 6 fold increase in kidney size, resulting in a KSI of 2.17 (Table 2). A comparison of spiggin levels in non-spawning male and female kidneys did not show any significant difference (p > 0.05), whereas the pre-spawning males showed an increase of spiggin levels (p < 0.01) when compared to both non-spawning males and non-spawning females (Fig. 5). This correlates well with the observed increase in KSI in pre-spawning sticklebacks. The total kidney weight in males was increased from about 5.8 mg in non-spawning fish to 35.7 mg in pre-spawning fish and the total amounts of spiggin in male kidneys was increased approximately 15 times, from 2.4 nmol to 36.1 nmol (Fig. 5a). The total amounts of spiggin per mg kidney in males increase from an average of 0.40 nmol/mg kidney in non-spawning males to 1.01 nmol/mg kidney in pre-spawning males (Fig. 5b).

**Table 2:** Body weight, kidney weight and KSI of male and female stickleback.

	Body weight (g)	Kidney weight (mg)	KSI
Non-spawning females (n = 5)	1.90 ± 0.32	14.1 ± 3.5	0.76 ± 0.24
Non-spawning males (n = 6)	1.23 ± 0.11	5.8 ± 1.5	0.48 ± 0.13
Pre-spawning males (n = 5)	1.69 ± 0.12	36.5 ± 7.9	2.17 ± 0.52

**Figure 6:**
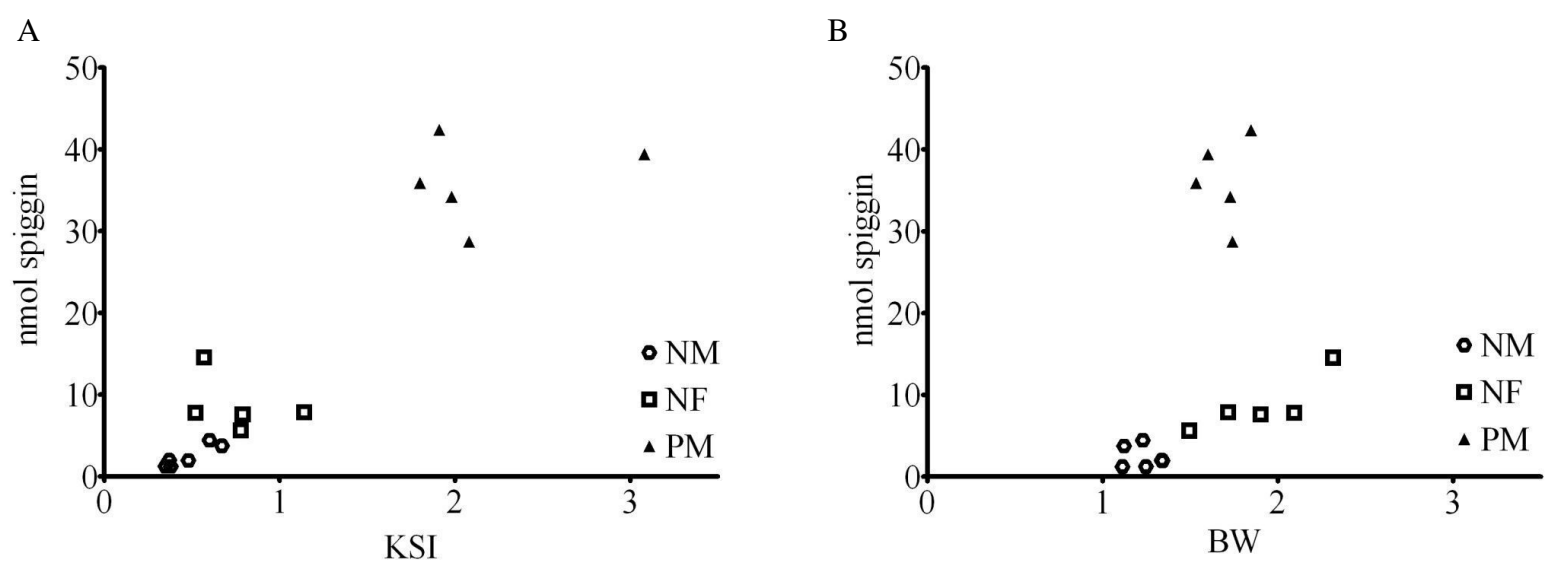
**Correlation of spiggin levels to kidney somatic index and body weight**. **A**. The total spiggin content (nmol) showed strong correlated to KSI. (p < 0.0001, r^2 ^= 0.95) **B**. The total spiggin content (nmol) showed no correlation to BW.

The total spiggin content correlated well with the KSI values and grouped the non-spawning males and females together, while the pre-spawning males were clearly separated based on both spiggin content and KSI (Fig 6a). On the other hand, a comparison of total spiggin content in comparison to body weight (BW) did not separate pre-spawning males from the other groups (Fig. 6b), indicating that it is the KSI rather than BW that correlates with spiggin induction.

**Figure 7:**
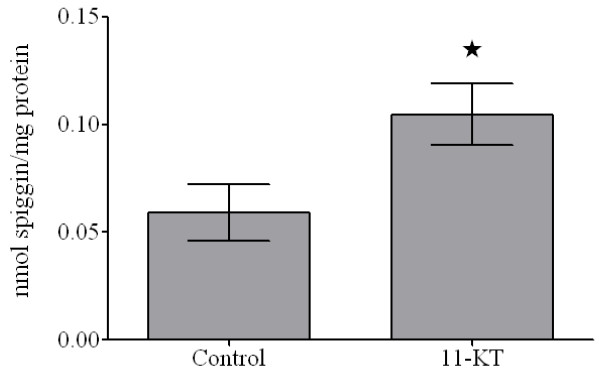
**Spiggin induction in 11-KT exposed fish**. Three-spined sticklebacks were exposed to 5 μg 11-KT/kg BW for three days. Renal spiggin levels were measured using the HRD antibody. Five animals were used for the control group while 4 animals were used for the exposed group. *, Statistically significant difference from the control (p < 0.05).

To further determine if the spiggin antibodies, developed against synthetic peptides, were specific for spiggin, sticklebacks were exposed to 11-KT (5 mg/kg BW) for 3 days and the spiggin levels in the urinary bladder was determined. Determination of absolute spiggin levels was performed using the HRD antibody and showed that the renal spiggin levels increased from 58.9 ± 26.4 pmol/mg protein to 104.5 ± 28.6 pmol/mg protein (Fig. 7), which is a 1.73 ± 0.26 increase. When using the KTK antibody for detection, the induction was found to be 1.79 ± 0.16 (data not shown).

**Figure 8:**
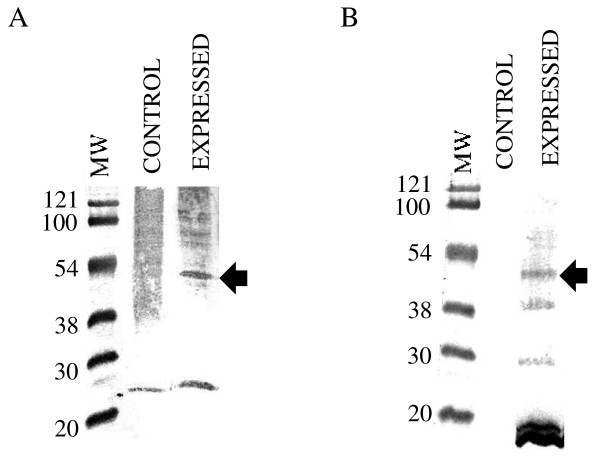
**Spiggin γ expression in cell and tissue lysates was examined by Western blot**. A. Detection of recombinant spiggin γ protein using the polyclonal native spiggin antibody; B. detection of His-tagged proteins using anti-His-tag antibody. MW – molecular weight markers (kDa); Control – bacterial lysate prior to IPTG addition; Expressed – bacterial supernatant following IPTG addition. The arrows indicate the position of spiggin γ protein.

In order to develop a universal standard for spiggin antibody detection we proceeded to clone the spiggin γ ORF into an expression vector for subsequent expression and detection. A theoretical calculation of the physical properties of spiggin γ showed that the protein had a theoretical molecular weight of approximately 54.5 kDa. Since spiggin has an ability to multimerize due to cystein bridge formation [2], the lysate preparation and analyses were performed under reducing conditions in the presence of 2-mercaptoethanol. Western blot analysis, using the spiggin antibody, confirmed the presence of spiggin γ in the lysis mixture. Spiggin γ was detected in the soluble fraction of bacterial lysate on Western blot with both the spiggin antibody (fig. 8a) and the anti-his-tag antibody (fig. 8b). Spiggin γ appears at a molecular weight corresponding to approximately 50 kDa.

## Discussion

During breeding male stickleback use the spiggin protein as an underwater adhesive to build their nest. The physical properties of spiggin protein render it extremely difficult to purify as multimerization of the protein results in aggregate formation and precipitation. On the other hand, spiggin would be a valuable tool to be able to measure, since it is one of the few proteins that so far are known to be under androgenic control and therefore could be used as a measurable parameter for androgen exposure.

This paper presents the development of sensitive quantitative antibody based spiggin assays useful for the detection of androgenic and anti-androgenic substances in the environment using three-spined stickleback as a model. Furthermore, it describes the use of recombinant spiggin γ as a standard for spiggin immunoblotting. The isoform composition of expressed spiggin protein complexes may vary in kidney and in urinary bladder [2]. Since the spiggin isoforms share the amino acids corresponding to the spiggin γ protein (Fig. 1), this isoform was chosen as a good candidate to use as a standard. The differences in the region for which the synthetic peptide KTK was raised is shown, with spiggin 2 and 3 lacking part of the sequence, whereas the sequence for the synthetic peptide HRD is present in all the sequences. The polyclonal native spiggin antibody used in the present study exhibits a high specificity as observed by ELISA and Western blot analysis. The ELISA showed that the spiggin antibody cross-reacts with the native adhesive protein in a linear range and in a concentration-dependent manner (Fig. 2). Spiggin polyclonal antibody against the native protein cross-reacted with native spiggin and produced several bands of different molecular weigh on the Western blot (Fig 4b). These bands correspond well with the expected sizes of the individual subunits and multimerized spiggin. Thus, the polyclonal antibody directed against native spiggin is able to cross-react with all spiggin subunits on Western blot analysis and also detects spiggin in ELISA analysis. Detection of native spiggin from the urinary bladder indicated a high level of multimerization with a strong band around 122 kDa and additional bands around 70 kDa and 52 kDa. In the kidney from female fish the protein is less abundant than in the males, including the multimerized isoforms. As in the males, bands corresponding to the sizes of all spiggin isoforms were detected in the kidney and the urinary bladder. (Fig. 4b). In contrast to males no multimerized spiggin could be identified in the urinary bladder of female stickleback. In male kidney there are bands corresponding to 230 kDa, 120 kDa, 100 kDa and 70 kDa. While the 230 kDa band corresponds to multimerized spiggin isoforms, the other bands correspond well with the calculated molecular weights of individual spiggin isoforms (Table 1).

The use of a synthetic peptide standard allows for quantification of spiggin and we have shown that the HRD peptide can be used in a competitive ELISA for detection of spiggin in methyltestosterone induced male and female three spined sticklebacks [8]. In this study both peptide antibodies were shown to be able to identify and quantify spiggin in biological samples. By using KTK and HRD in an ELISA it was found that a three day 11-KT exposure gave rise to a 1.73 and 1.79 spiggin increase respectively, indicating that the two antibodies can be used individually to measure androgen induced spiggin production.

Due to the native spiggin proteins highly hydrophobic properties, which are instrumental for its use by stickleback males as underwater glue, it poses challenges in handling and detection. Therefore, the use of recombinant V5/His6-tagged spiggin γ as a standard for spiggin measurements offers an alternative in order to obtain quantitative measurements of spiggin in biological samples. Addition of V5/His6 epitopes to recombinant proteins has shown to increase their solubility [9] and recombinant spiggin can be produced in bacteria in necessary amounts and in a short period of time which makes it highly available.

On the western blot, the spiggin antibodies against the native protein and antibodies against the synthetic peptides cross-reacted with the recombinant protein in bacterial cell lysate enabling detection of a recombinant protein of approximately 50 kDa. The molecular weight of the recombinant protein on Western blot was less than the theoretical protein weight which is in agreement with previously reported results [2]. Since *E coli *lack glycosylation properties, discrepancies in theoretical and experimental molecular weight for the recombinant spiggin could be explained by the physical properties of the spiggin. The high hydrophobicity of spiggin might also contribute to this phenomenon due to higher affinity to SDS [10].

Spiggin shows great potential as a screening tool for endocrine disruptors, and has been used for this purpose in kidney cell cultures [11], tissue slice cultures [12] and in whole animal exposures [13]. However, there are many factors in the environment that might affect the production of the adhesive protein. For instance, parasite harbouring fish should be excluded to avoid errors, as infection of *Schistocephalus solidus *in male three-spined stickleback result in reduced levels of spiggin in the kidney [14]. The correlation between total spiggin content in all three groups with KSI suggests that KSI measurements could be used as an initial rapid screening method to detect possible androgenic disturbances.

Due to the adhesive and multimerization properties of spiggin it is of interest to develop methods to prevent polymerisation of both native and recombinant protein upon purification. We have tested the effets of saline solution as solvent to simplify handling of samples. Adjustments of pH was not found to improve the solubility of spiggin whereas a saline solution kept spiggin soluble for several days in +4°C (data not shown). Even so, the positive effect of salinity on spiggin soulubility could not be used in the ELISA procedures since the small volumes of the samples added to the buffer used in ELISA results in low final concentrations of salt and a rapid mutlimerization of the protein in the wells.

The seasonal cycling in plasma levels of 11-ketotestosterone in the male three-spined stickleback show an marked increase in May with a peak from June to July, this peak is also mirrored in the kidney epithelial height, (KEH), which increases from approximately 15 μm to 40 μm in the spawning season [15,16]. Seasonal variations in KSI showed an increase from March to April, peaking in May, followed by a decrease in June to July [17]. Measurements of spiggin in conjunction with measurements of KSI would enable trend monitoring of local populations over time to obtain data on possible increases in accumulation of endocrine disrupting substances, and the appearance of previously undetected environmental androgens. The cloning and characterization of the stickleback nuclear androgen receptor [18] will be a useful tool for future work on identification of androgenic substances in the aquatic environment.

In the present study we have characterized three spiggin antibodies and demonstrated that they are highly specific and able to detect basal levels of spiggin in both male and female fish. All three antibodies were able to detect protein corresponding to all the known sizes of spiggin, suggesting that they will give a total spiggin value when used in ELISA assays. We have also developed two quantitative spiggin ELISA using peptide standards and have shown that posibility of producing recombinant spiggin protein for use as a standard with native spiggin antibodies.

## Competing interests

The authors declare that they have no competing interests.

## Authors' contributions

HB carried out initial antibody screening, the 11-KT induction study and helped to draft the manuscript. NS cloned and expressed spiggin γ, performed immunoassays on recombinant spiggin and helped to draft the manuscript. HL carried out the immunoassays. EH participated in the whole animal experiments. JK participated in the characterization of the antibodies. PEO conceived the study, designed the antibodies and drafted the manuscript. All authors contributed to the writing of the manuscript and approved the final manuscript.
